# Protective Effects of N-Acetyl Cysteine against Diesel Exhaust Particles-Induced Intracellular ROS Generates Pro-Inflammatory Cytokines to Mediate the Vascular Permeability of Capillary-Like Endothelial Tubes

**DOI:** 10.1371/journal.pone.0131911

**Published:** 2015-07-06

**Authors:** Chia-Yi Tseng, Jing-Fen Chang, Jhih-Syuan Wang, Yu-Jung Chang, Marion K. Gordon, Ming-Wei Chao

**Affiliations:** 1 Department of Biomedical Engineering, College of Engineering, Chung Yuan Christian University, Zhongli district, Taoyuan city, Taiwan; 2 Center of Nanotechnology, Chung Yuan Christian University, Zhongli district, Taoyuan city, Taiwan; 3 Department of Bioscience Technology, College of Science, Chung Yuan Christian University, Zhongli district, Taoyuan city, Taiwan; 4 Joint program of Toxicology, Rutgers University, Piscataway, New Jersey, United States of America; University of Illinois at Chicago, UNITED STATES

## Abstract

Exposure to diesel exhaust particles (DEP) is associated with pulmonary and cardiovascular diseases. Previous studies using *in vitro* endothelial tubes as a simplified model of capillaries have found that DEP-induced ROS increase vascular permeability with rearrangement or internalization of adherens junctional VE-cadherin away from the plasma membrane. This allows DEPs to penetrate into the cell and capillary lumen. In addition, pro-inflammatory cytokines are up-regulated and mediate vascular permeability in response to DEP. However, the mechanisms through which these DEP-induced pro-inflammatory cytokines increase vascular permeability remain unknown. Hence, we examined the ability of DEP to induce permeability of human umbilical vein endothelial cell tube cells to investigate these mechanisms. Furthermore, supplementation with NAC reduces ROS production following exposure to DEP. HUVEC tube cells contributed to a pro-inflammatory response to DEP-induced intracellular ROS generation. Endothelial oxidative stress induced the release of TNF-α and IL-6 from tube cells, subsequently stimulating the secretion of VEGF-A independent of HO-1. Our data suggests that DEP-induced intracellular ROS and release of the pro-inflammatory cytokines TNF- α and IL-6, which would contribute to VEGF-A secretion and disrupt cell-cell borders and increase vasculature permeability. Addition of NAC suppresses DEP-induced ROS efficiently and reduces subsequent damages by increasing endogenous glutathione.

## Introduction

Diesel exhaust particles (DEP) are associated with induction and exacerbation of cardiovascular disorders through the production of harmful free radicals and initiation of pro-inflammatory responses [1–3]. These events contribute to the resulting adverse health effects of DEP. Endothelial dysfunction can be caused by diesel-induced mechanisms [4, 5]. Meanwhile, vasoconstriction has been found in humans who inhaled ambient PM_2.5_ (particulate matter ≤ 2.5 μm) for 2 h [6], and impaired vasodilatation appears 24 h after 1 h of DEP inhalation [5]. Moreover, the water content of the mouse lung which causes epithelial fibrosis and disrupts vascular function (increased 24 h after intratracheal instillation of DEPs or DEP extracts), which has been suggested to cause an increase in pulmonary endothelial permeability [7].

DEP have been found to enter into the circulation and may have a direct effect on endothelial permeability. *In vitro*-assembled capillary-like endothelial tubes are used to model the translocation of particles into capillaries [8]. A previous study demonstrated, using confocal microscopy, that DEP affect the permeability of endothelial tubes. The adherens junction component, VE-cadherin, moves from cell-cell junctions to intracellular locations, and DEP deposit in the cytoplasm of cells and the luminal space of tubes. Disruption of VE-cadherin endothelial barrier integrity has also been shown to alter vascular permeability [9, 10]. The pulmonary endothelium acts as a semipermeable barrier, and the integrity of the barrier is necessary for efficient pulmonary function [11]. Furthermore, recent results have suggested that DEPs function by changing the levels of its effector, H_2_O_2_, which triggers Nrf2 translocation from the cytoplasm to the nucleus [12, 13]. Downstream heme oxygenase (HO)-1 is then upregulated to facilitate the anti-oxidative stress response in the endothelium [14–17]. HO-1 also functions to induce vascular permeability and contributes to the secretion of vascular endothelial growth factor A (VEGF-A) [18]. VEGF-A, also called vascular permeability factor (VPF), has been shown to induce vascular permeability [19, 20]. Upon exposure of *in vitro* capillary tube cells to DEPs, the VE-cadherin/VEGF receptor 2 (VEGF-R2) complex on the cell membrane dissociates [12]. Partial internalization of VE-cadherin and discontinuity of the cell-cell border are also induced following these junctional alternations [12, 21]. Moreover, these events cause endothelial junctions to become disrupted and may explain how VEGF-A initiates vascular permeability following inhalation of DEP.

DEP exposure damages the alveolar endothelia and sequentially induces pro-inflammatory responses [22, 23], in which cytokines function to enhance vascular permeability and cause transmigration of inflammatory cells [24, 25]. Among these pro-inflammatory cytokines, tumor necrosis factor (TNF)-α and interleukin (IL)-6 have been investigated most widely in studies of DEPs [26, 27]. Inhalation of DEPs for 24 h upregulates TNF-α and leads to accumulation of large amounts of TNF-α in human plasma [5]. This changes the expression of adhesion molecules on endothelial cells, facilitating the transmigration of neutrophils and thereby leading to changes in vascular permeability [28]. Furthermore, there is a positive correlation between vascular permeability and adherens junction integrity [22]. Nwariaku *et al* (2002) found that TNF-α-induced tyrosine phosphorylation of VE-cadherin, which permits regulation of microvascular permeability, increases the formation of intercellular gap formation [29]. IL-6 has also been shown to be directly involved in increasing monolayer endothelial permeability [30]. Maruo *et al* (1992) suggested that IL-6 induces increased endothelial permeability by rearranging VE-cadherin and altering the shape of endothelial cells. This implies that the endothelial cell-cell barrier may also be altered [31]. Importantly, when IL-6 is knocked down, vascular permeability is restored.

Although these cytokines are thought to play important roles in stimulating monolayer vascular permeability, the mechanisms mediating this process remain unclear in capillary-like tube cells, a biologically relevant model. Our group has reported that ROS is an inducer to contribute VEGF-A releasing [12]. Then DEP initiates mitochondrial oxidative stress generation, leading to cause ATP depletion and depolymerization of actin cytoskeleton [32]. Subsequently the particle might slip between the cells via redistributed VE-cadherin network, and travel in the circulation system [8]. The pathway through which DEP-induced oxidative stress triggers VEGF-A secretion and facilitates permeability has been observed in various cell types [23, 33–35]; however, whether pro-inflammatory cytokines also function in induction of VEGF-A in the tube-like endothelial model remains unknown. Therefore, in this study, we used an endothelial tube model, a system free of the confounding effects of inflammatory cells, to examine how cytokines might modulate endothelial permeability. We also evaluated the mechanisms mediating intracellular oxidative stress induction, endothelial pro-inflammatory responses, VEGF-A secretion, VE-cadherin distribution, and vascular permeability, and the protection of ROS scavenger N-acetyl cysteine (NAC).

## Materials and Methods

### Cell culture

HUVECs were cultured in Medium 199 with endothelial cell growth supplement (Millipore, Billerica, MA, USA), heparin (Sigma-Aldrich, St. Louis, MO, USA), and 10% FBS. HUVECs at passages 5–15 were used to assemble tubes on 10 mg/mL Matrigel, LDEV-free (BD Biosciences). Endothelial tubes were allowed to form for 12 h prior to adding DEPs. Well-characterized DEPs were collected from Dr. Masaru Sagai, Aomori University of Health and Welfare, Japan [1, 36]. Particles were prepared and used as previously described to create a suspension resulting in sizes of PM_2.5_, of which a portion was PM_0.1_ [8].

### Reagents

NAC was obtained from Sigma-Aldrich. TNF-α and IL-6 were purchased from R&D Systems, Inc (Minneapolis, MN, USA). SnPP (protoporphyrin IX) was purchased from Frontier Scientific, Inc. (Logan, UT, USA).

### Determination of total, reduced and oxidized glutathione levels

Total glutathione content of the cell extracts was assessed using a “total glutathione assay kit” (CS0260-1KT, Sigma-Aldrich) based on a kinetic assay in which catalytic amounts of GSH caused a continuous reduction of 5,5-dithiobis-(2-nitrobenzoic) acid (DTNB) at 412 nm. For oxidized glutathione (GSSG) determination, reduced glutathione (GSH) was inactivated by the addition of 2-vinylpyridine in the presence of triethanolamine. GSH content was calculated using the equation of GSH = (Total GSH– 2 x GSSG). Quantification was achieved by parallel measurements of GSH or GSSG standards, and results were expressed as nmol/ mg protein [37].

### Quantification of extracellular and intracellular ROS generation

Extracellular and intracellular ROS detection studies were performed using a Cm-H2DCFDA ROS detection kit (Invitrogen, Grand Island, NY, USA). For cell-free ROS detection, aliquots of 100 μL serum-free medium containing each concentration of DEPs with or without NAC were pipetted into 96-well plates and mixed with 10 μL HBSS containing Cm-H_2_DCFDA (final concentration 25 μM), activated by pre-incubation at 37°C for 30 min [37]. ROS generation was measured every 10 min up to 2 h with a microreader at 490/530 nm. Samples treated with 100 μM H_2_O_2_ were used as positive controls. For detection of intracellular ROS, after the treatments, they were washed with PBS and treated with fresh serum-free medium containing Cm-H_2_DCFDA (final concentration 25 μM). Samples were then measured for 60 min after Cm-H_2_DCFDA was activated. ROS levels generated by 1.5 × 10^5^ viable treated cells were expressed as the percentage of ROS produced by an equal number of viable negative control cells.

### Determination of Cytotoxicity

DEP-induced cytotoxicity in endothelial tubes was evaluated using the CytoTox- Homogeneous Integrity Assay Kit (Promega), a method that measures cytosolic lactate dehydrogenase (LDH) released into medium when cells are lysed. After the treatments, cells were collected by centrifugation and lysed for 1 h in 200 μL lysis solution following the manufacturer’s instructions. The value of LDH was measured at 490 nm. Untreated cultures were presented as the negative control, defining the maximum amount of LDH potentially released.

### Measurement of IL-6 and TNF-α levels

TNF-α and IL-6 were evaluated using a Human TNF-α, IL-6, and IL-1β Ready-SET-Go! ELISA Kits (eBioscience, San Diego, CA, USA), following the manufacturer’s instructions. Briefly, triplicate 6-well plates were seeded with 1.5 × 10^5^ HUVECs/well, and after tubes were formed, various concentrations of DEPs (0, 1, 10, or 100 μg/mL) with or without NAC (10 mM) were added for 24 h. Next, 200 μL medium was collected and added to the wells, which were precoated with cytokine “capture” antibodies. The plates were incubated overnight at 4°C. The next day, the medium was aspirated, and wells were washed with 2% normal goat serum containing 0.02% NaN_3_ in PBS for 1 h at room temperature. After aspirating off the wash solution, a biotin-conjugated “detecting” primary antibody targeting either IL-6 or TNF-α was added, and plates were incubated at room temperature for 1 h. The supernatant was then aspirated off, and the wells were washed several times. Avidin conjugated-horse radish peroxidase (HRP) was added, and plates were incubated for 1 h at room temperature. Finally, substrate solution (tetramethylbenzidine) was added for 15 min at room temperature, and the plates were read at 450 nm. The levels of IL-6 and TNF-α were determined by comparison to a standard curve, which was prepared by analyzing 2-fold serial dilutions of each cytokine. For this, plates whose wells were precoated with capture antibodies were used, and the same procedure as outlined above was carried out using the serial dilutions rather than the sample medium.

### Vascular permeability assay

To analyze vascular permeability, we used the method described previously by Chao *et al* (2012) [12]. Test sample wells were coated with Matrigel, and cells were then added and allowed to form tubes before exposure to the chemicals for 24 h. After incubation, free DEPs were washed away with PBS, and medium (1 mL/well) containing FITC-conjugated dextran at a final concentration of 1 mg/mL was added into the culture dishes. After a 4-h incubation, the endothelial tubes were washed twice with PBS and collected by scraping the wells. Samples were gently pipetted up and down to prepare cell extracts. After BCA quantification, samples were all adjusted to 4 mg/mL protein. To ascertain whether FITC had permeated the tube structures, 200 μL of each lysate was loaded into a 96-well plate for determination of the RFU of each sample. Readings were performed using an HTS 7000 Plus Bio Assay Reader at 490 nm (Perkin Elmer Life Sciences). The “Matrigel only” samples were treated identically as the blank.

### Total RNA extraction and quantitative polymerase chain reaction (qPCR) analysis

Total RNA was isolated from *in vitro* HUVEC capillary tubes using TRIzol reagent (Sigma-Aldrich). RNA was quantified and reverse transcribed into cDNA using oligo-dTs and a Superscript Synthesis system kit (Invitrogen). To assess the quantity of endogenous HO-1 and VEGFR2 in *in vitro* HUVEC capillary tubes, the primers used for the amplification were as follows: *HO-1*, 5′-AGGCCAAGACTGCGTTCCT-3′ and 5′-GGTGTCATGGGTCAGCAGC-3′ (Hirai *et al*., 2003); *VEGFR2*, 5’-GCTCAAAAAGATTGCCCAGA -3’ (forward) and 5’- GCGGTAGAGCTGCTTGAACT-3’ (reverse). The primers were used at a final concentration of 300 nM. SYBR Green (Applied Biosystems, Foster City, CA, USA) was employed for fluorescence-based quantification of cDNA by real-time qPCR using an ABI Prism 7000 thermal cycler. The qPCR product was evaluated and normalized to 18S ribosomal mRNA levels and the negative control.

### Western blotting

For preparation of whole cell lysates, tube cells were collected and sonicated for 1 min in 1 mL lysis buffer. The cell lysates were cleaned by centrifuging at 10,000 × *g* for 10 min, and the supernatant was collected. Nuclear protein extracts were prepared from endothelial tube cells using an adapted 1-h minipreparation technique [12]. Briefly, cells were collected and sonicated for 1 min in a relatively low-salt lysis buffer and then centrifuged for 30 s at 2500 rpm. The supernatant, which contained the nuclei, was next incubated for 5 min on ice and then centrifuged for 5 min at 5000 rpm. The pelleted nuclei were resuspended in a higher-salt lysis solution and incubated on ice for 20 min. Insoluble nuclear debris was pelleted by centrifugation for 30 s. The protein concentrations of the whole cell extract and nuclear extract were determined using BCA assays (Pierce), and the samples were stored at −80°C. Sodium dodecyl sulfate polyacrylamide gel electrophoresis (SDS-PAGE) was then carried out using 20 μg protein per lane.

For detection of secreted proteins, the supernatant/medium of HUVEC capillary tubes was collected after treatment with DEPs. A total of 40 μL per sample of supernatant/medium was loaded onto SDS gels for electrophoresis after boiling at 95°C for 5 min. Coomassie blue was used as the loading control, and fresh medium was loaded as the negative control. Protein expression was normalized to the 150-kDa band in the loading control. Proteins separated electrophorectically on SDS gels were transferred to nitrocellulose membranes. Nonspecific reactivity of the membranes was blocked and the primary anti-HO-1, anti-TNF-α, anti-IL-6, anti-IL-1β, anti-IκB, anti-nuclear p65, anti-lamin B1, anti-VEGF-A, anti-VEGF-R2, anti-VE-cadherin, anti-β-actin, and anti-GAPDH antibodies were applied at appropriate dilutions at 4°C overnight. Secondary goat anti-mouse IgG or anti-rabbit IgG antibodies conjugated with HRP (Bio-Rad, Hercules, CA, USA) were used at 1:5000 dilution. Finally, the protein products were visualized on X-ray film using with chemiluminescent reagent containing luminol, a substrate of HRP.

### Immunofluorescence microscopy

After treatment, the tube cells were fixed with 4% paraformaldehyde and blocked by 2% normal goat serum. Sequentially, the tube cells were incubated with primary anti-VEGF-R2 and VE-cadherin antibody at a 1:100 dilution and then exposed to the secondary antibody labeled with Alexa 488 (Invitrogen) at a 1:200 dilution. Slides were covered with Prolong Gold anti-fade mounting media (Invitrogen) with DAPI and stored at 4°C overnight. Images were observed at 400× magnification on an IX51 Olympus Microscope.

### Statistics

For statistical analysis, each experiment was performed at least 3 times, and samples were always run in triplicate. The results were expressed as means ± standard deviations (SDs) and were analyzed using multiple t-tests. Differences with *P*-values of less than 0.05 were considered statistically significant.

## Results

### DEP-induced intracellular ROS generation and cytotoxicity

**Fig 1:**
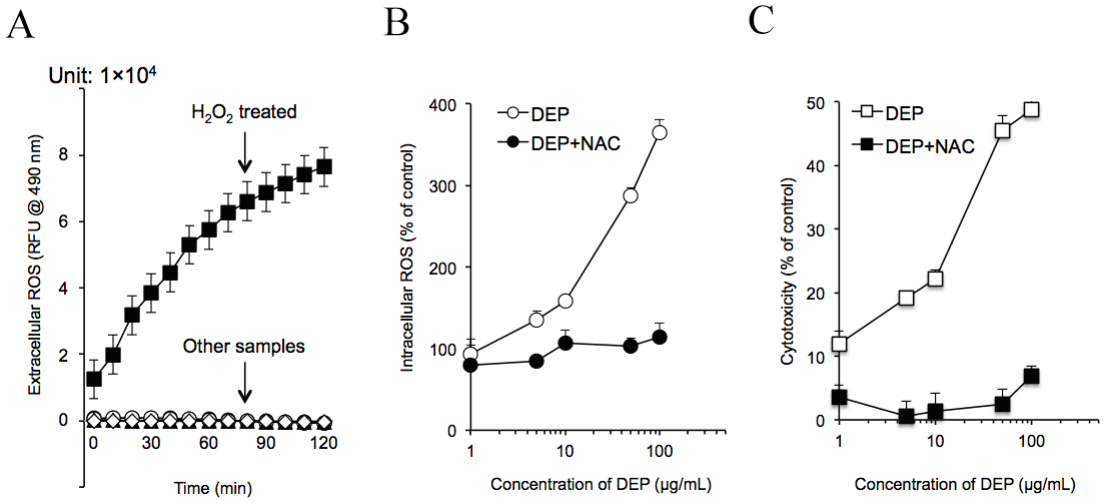
DEPs induced intracellular ROS generation in capillary-like tube cells and caused cytotoxicity. (A) DEPs were incubated in the cell-free system for 2 h. Cm-H_2_DCFDA assays were then carried out. ROS production is shown for each sample. (B) HUVECs were exposed to DEPs ± NAC for 24 h. Intracellular ROS is shown. (C) Tube cells were exposed to DEPs with or without the addition of NAC. Cytotoxicity was measured as described in the Methods.

Chao *et al* (2012) demonstrated that tube cells exposed to 1 μg/mL DEPs generates H_2_O_2_ (0.18 nmol). At 10 and 100 μg/mL concentrations of DEPs, production of H_2_O_2_ in tube cells was increased to 0.51 and 2.05 nmol, respectively [12]. Thus, cytotoxicity might be a consequence of exposure to DEP-induced oxidative stress. However, whether these free radicals are generated from DEPs before they contact the cells or after they are taken up by cells and induce sequential intracellular oxidative damage remains unclear. Therefore, we used Cm-H_2_DCFDA to evaluate this pathway. As shown in Fig 1A, changes in relatively fluorescence units (RFU) could be used to demonstrate the level of DEP-induced ROS in the cell-free model. From these data, the increased RFU was observed only in the H_2_O_2_-containing sample with a time-dependent manner. Other samples containing DEPs (10 or 100 μg/mL) ± NAC (10 mM) showed no detectable changes in RFU. In contrast, Cm-H_2_DCFDA analysis showed that DEP induced intracellular ROS generation in tube cells in a dose-dependent manner (Fig 1B). Addition of NAC, oxidative stress was completely blocked. Fig 1C shows cytotoxicity curves for HUVEC tube cells treated with DEPs (1, 5, 10, 50, or 100 μg/mL) ± NAC. Cytotoxicity in tube cells exposed to 1 μg/mL DEP reached 12%; additionally, 5 and 10 μg/mL DEPs induced cell death in 19% and 22% of cells, respectively. Higher doses of DEPs (50 and 100 μg/mL) also induced greater cell death (45% and 49%, respectively). Thus, cytotoxicity was increased in a dose-dependent manner, similar to the results shown in Fig 1B, suggesting that intracellular ROS might affect cell survival. These experiments not only confirm the results generated by Chao *et al* (2012), but also suggest that cytotoxicity is directly caused by the free radicals generated in tube cells [12].

### NAC provides increase of GSH/GSSG redox ratio in capillary-like tube cells

It is important to confirm whether NAC stimulates GSH up-regulation in the HUVEC tube cells. As shown in Table 1, total GSH and GSSG levels showed marked dose-dependent increases in the DEP treated samples vs. control, which means DEP-induced ROS oxidized GSH and converted it to GSSG. NAC supplementation provided marked decreases in GSSG levels compared to DEP group. Besides, DEP+NAC exposure supplied none significant increases in total GSH level; however, GSSG level had an obviously reduction. Additionally, GSH/GSSG redox ratio revealed a significant difference when compare to DEP and DEP + NAC samples from 2.13, 4.08, 4.32, and 5.13 fold of control at 0, 1, 10, 100 μg/mL, respectively. It gives us an evidence to suggest that NAC might provide dramatic increase of intracellular GSH level to against DEP-induced oxidative stress.

**Table 1:** Cytoplasmic Total, Oxidized and Reduced Glutathione Levels and GSH/GSSG Redox Ratio in the tube cells treated with DEP ± NAC. Total GSH, total glutathione; GSSG, oxidized glutathione; GSH, reduced glutathione. Values are given as mean ±SD of n = 3 experiments and duplicate measurements. The concentrations of DEP used are 1, 10, and 100 μg/mL, NAC is 10 mM. DEP (0 μg/mL) presents as the negative control.

		Total GSH	GSSG	GSH	GSH/GSSG
		(U/mg protein)	(U/mg protein)	(U/mg protein)	Redox Ratio
DEP (μg/mL)	0	15.2 ± 0.86	1.5 ± 0.06	13.4 ± 1.27	8.93
	1	16.5 ± 0.95	3.6 ± 0.12^a^	12.5 ± 1.43	3.47^a^
	10	18.6 ± 1.23^a^	4.6 ± 0.48^a^	11.8 ± 1.89	2.57^a^
	100	21.8 ± 1.91^a^	7.6 ± 0.8^a^	13.1 ± 1.15	1.72^a^
DEP (μg/mL)	0	20.1 ± 2.09^b^	0.9 ± 0.01^b^	17.1 ± 1.63^b^	19^a^ ^b^
plus NAC	1	21.4 ± 1.89^b^	1.3 ± 0.11^b^	18.4 ± 1.41^b^	14.15^a^ ^b^
	10	21.9 ± 2.11	1.7 ± 0.09^b^	18.9 ± 1.26^b^	11.12^a^ ^b^
	100	22.8 ± 2.47	2.3 ± 0.07^a^ ^b^	20.3 ± 1.71^b^	8.82

^a^ is significantly different from the samples to DEP (0 μg/mL) in upper column or to NAC+DEP (0 μg/mL) in lower column (p < 0.05)

^b^ is significant difference between DEP and NAC+DEP samples (p < 0.05).

### DEP-induced ROS trigger secretion of the endogenous cytokines TNF-α and IL-6

**Fig 2:**
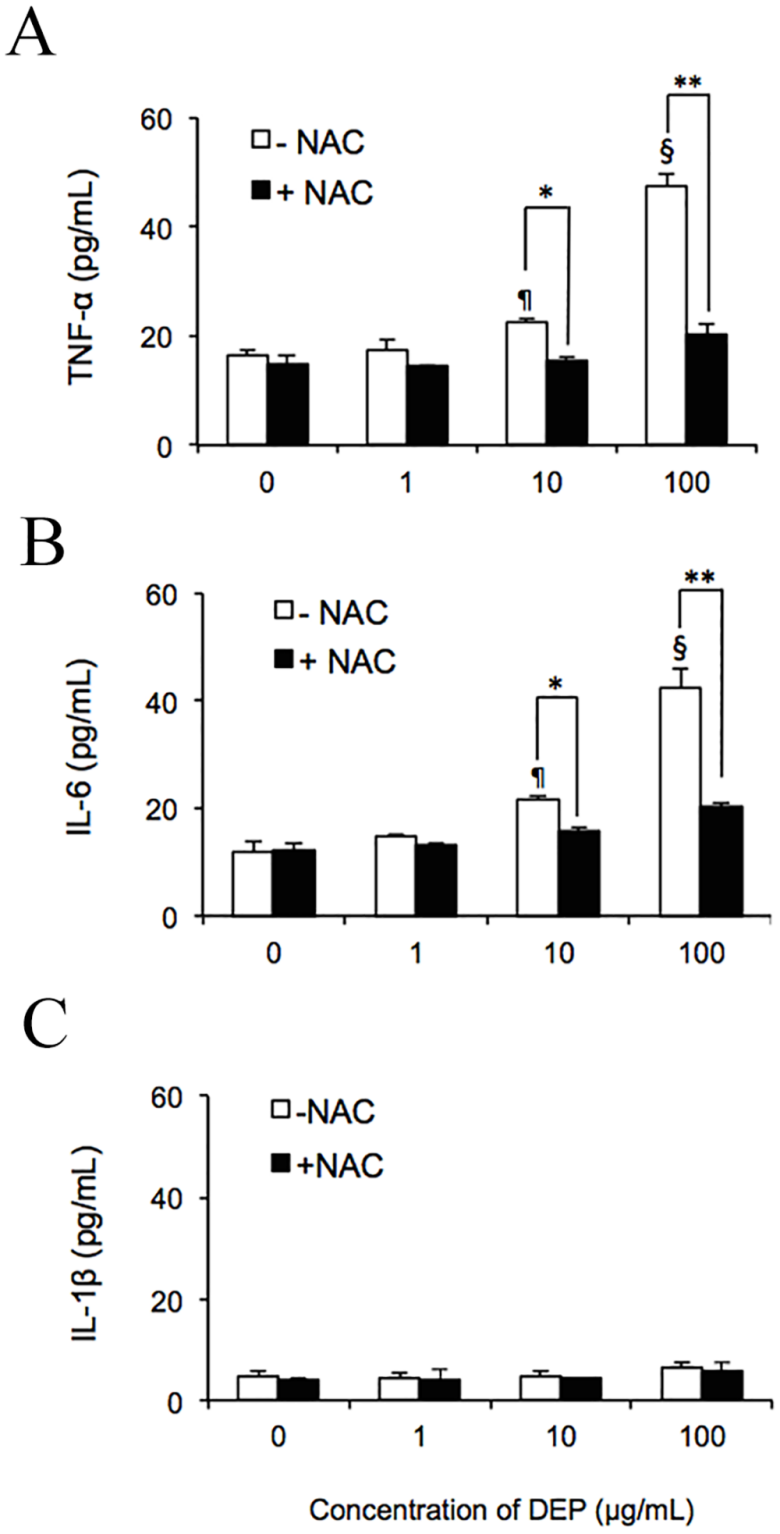
DEP-induced ROS promoted secretion of TNF-α and IL-6 by capillary-like tube cultures *in vitro*. After exposing endothelial tubes to 1, 10, or 100 μg/mL DEPs with or without NAC, medium was collected for evaluation by ELISA. Concentrations of TNF-α (A), IL-6 (B), and IL-1β (C) were measured and were quantified according to comparison with a standard curve. ¶ and § indicate significant differences (*P* < 0.01 and *P* < 0.001) between DEP-treated samples and the untreated control (0 μg/mL). Significance was reached at *p < 0.05 and **p < 0.01 in samples treated with 10 and 100 μg/mL DEPs compared to the same amount of DEPs plus NAC. Values represent means ± SDs (n = 6). Statistical analysis was carried out using the Student’s t-test.

**Fig 3:**
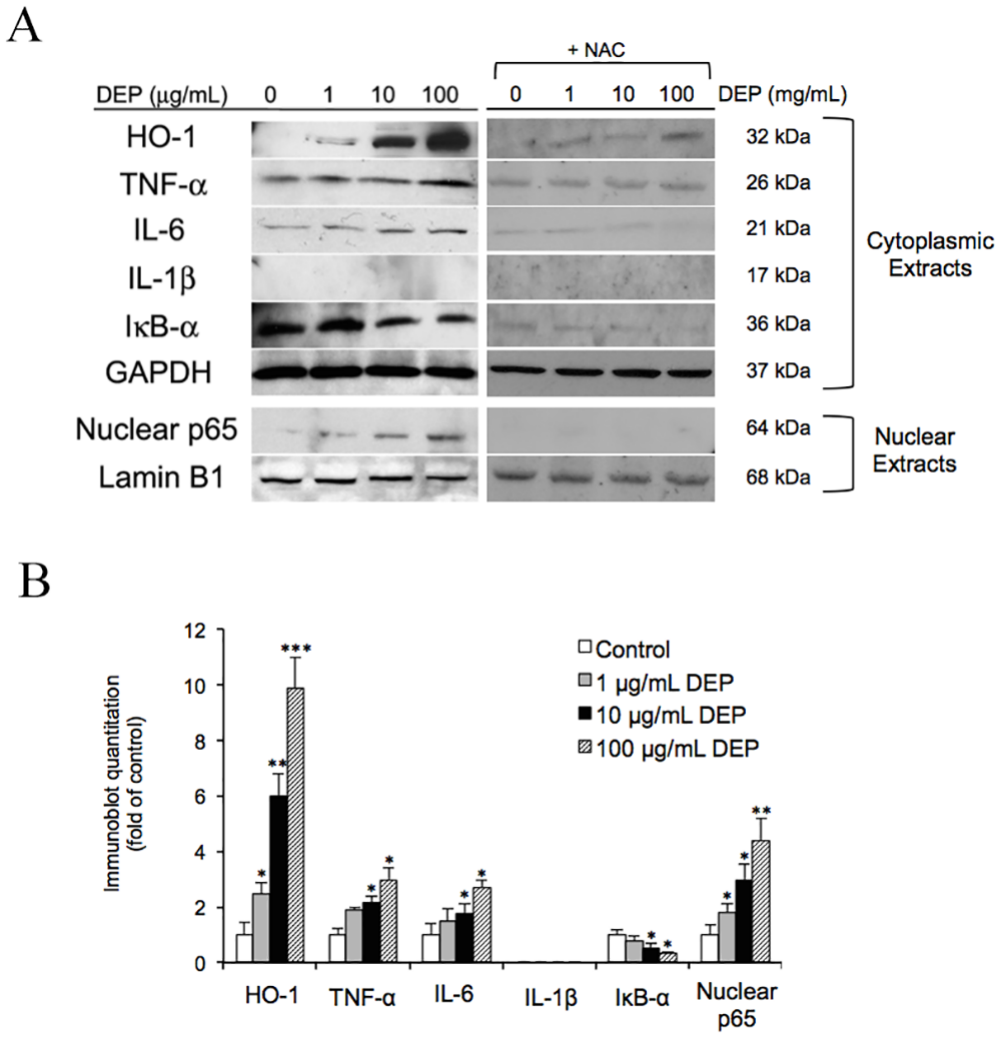
DEPs affected the expression of intracellular proteins. (A) After a 24-h exposure to DEPs ± NAC, endothelial tube cells were lysed, and cytoplasmic and nuclear proteins were extracted for western blot analysis. The 8 lanes of the immunoblot represent samples exposed to different concentrations of DEPs and DEPs+NAC. Equal loading of cytoplasmic and nuclear proteins was confirmed with GAPDH and Lamin B1 immunoreactivities, respectively. (B) Semiquantitative analysis of the band intensities. Significance was reached at *p < 0.05, **p < 0.01, and ***p < 0.001, as analyzed using Student’s t-test.

The respiratory system and capillaries have been shown to respond to DEP exposure by secreting the pro-inflammatory cytokines TNF-α and IL-6 [7, 38, 39]. To examine whether DEP-induced ROS triggered the release of these cytokines from *in vitro* endothelial tubes and NAC reduces this activity, cultures were treated for 24 h with 1, 10, or 100 μg/mL DEPs ± NAC. As determined by comparison with an enzyme-linked immunosorbent assay (ELISA)-generated standard curve, negative control HUVEC tubes released 16.6 pg/mL TNF-α (Fig 2A) and 11.8 pg/mL IL-6 (Fig 2B) into the medium. No significant difference was observed in cytokine levels when cells were treated with 1 μg/mL DEPs. However, at 10 μg/mL DEPs, 22.7 ± 0.6 pg/mL TNF-α and 21.8 ± 0.6 pg/mL IL-6 was found in the medium. Moreover, after a 24-h exposure to 100 μg/mL DEPs, tube cells released 47.5 ± 2.3 pg/mL TNF-α and 42.4 ± 3.6 pg/mL IL-6, about 4 times that of the untreated control. The presence of NAC significantly suppressed the release of both cytokines. With this ROS scavenger, secretion was reduced to 20.4 ± 5.2 pg/mL TNF-α and 20.2 ± 0.84 pg/mL IL-6 in samples treated with 100 μg/mL DEPs, comparable to levels of these cytokines in cells treated with 10 μg/mL DEPs without the addition of NAC, strongly indicating that DEP-induced ROS mediated the secretion of these cytokines from endothelial tube cells. However, as shown in Fig 2C, no significant upregulation of IL-1β was observed in response to DEPs. Furthermore, as shown in Fig 3, immunoblotting results supported that DEPs dramatically increased levels of cytoplasmic HO-1 and slightly increased levels of TNF-α and IL-6. IκB-α was reduced in a dose-dependent manner following treatment with DEPs, while IL-1β was not observed. In nuclear extracts, nuclear p65 was elevated in response to DEP exposure. Addition of NAC significantly reduces HO-1, TNF-α, IL-6, IκB-α, and p65 expression. Quantification of the blots of DEP treated samples is shown in Fig 3B.

### TNF-α and IL-6 stimulated the release of vascular permeability factor (VPF)/VEGFA

**Fig 4:**
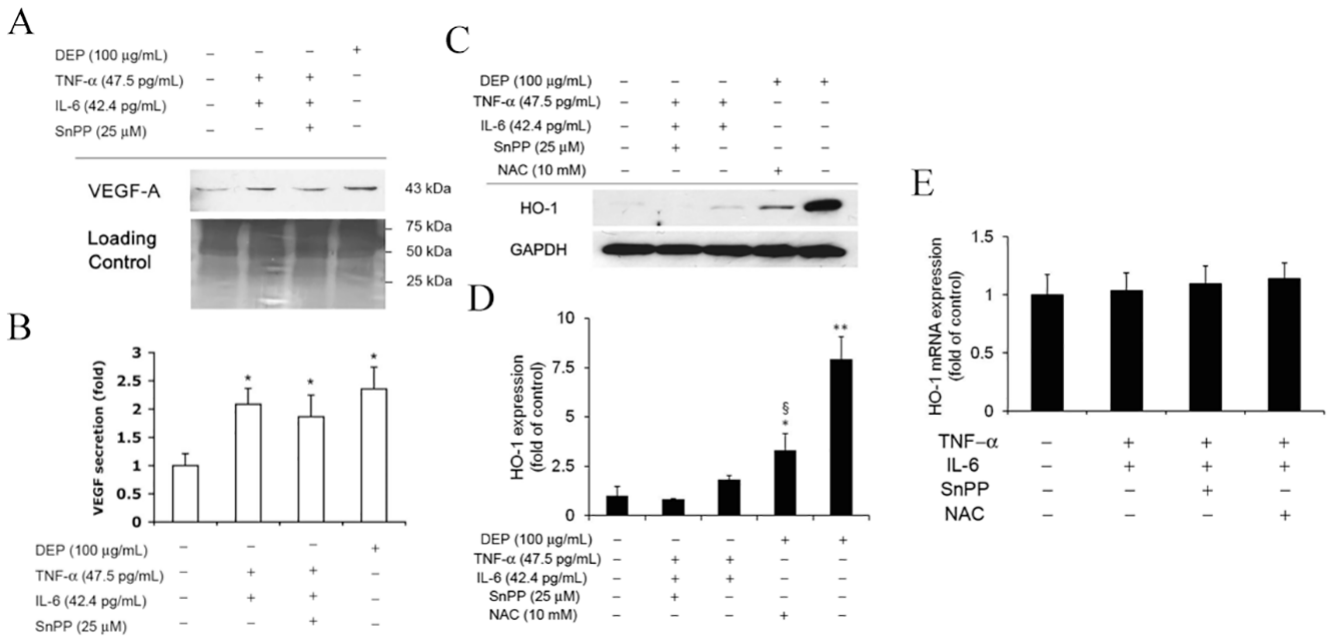
Addition of TNF-α and IL-6 triggered release of VEGF-A from endothelial tube cells. (A) The tube cells were treated with 47.5 and 42.4 pg/mL TNF-α and VEGF-A, respectively, and incubated for a 24 h. The medium was collected and subjected to SDS-PAGE. Half the gel was used for western blot analysis, probing with anti-VEGF-A antibodies, and the other half was identically loaded as a loading control, stained with Coomassie blue. (B) Semiquantitative analysis of the intensity of VEGF-A bands from the blot. Furthermore, TNF-α plus IL-6 independently stimulated HO-1 expression. (C) Analysis of HO-1 expression in HUVEC tube lysates was performed using immunoblotting. Equal protein loading was confirmed with GAPDH immunoreactivity. (D) Semiquantitative analysis of the intensities of HO-1 bands from the blot. § indicates *p* < 0.05 significance decrease from DEP (100 g/mL) to NAC+DEP (100 g/mL). (E) *HO-1* mRNA expression response to TNF-α and IL-6 was quantified using qPCR, with normalization to 18S. HO-1 expressed in the negative control was defined as 1. Values are expressed as the fold-change compared to the negative control (n = 3). Significance was reached at *p < 0.05 as analyzed using Student’s t-test.

HO-1 has previously been shown to induce the secretion of VPF/VEGF-A [18, 40, 41]. Chao *et al* (2012) demonstrated that DEPs upregulate HO-1 and stimulate VEGFA secretion into the culture media [12]. Because our previous experiment only investigated whether TNF-α and IL-6 were upregulated, we wondered whether these cytokines affected permeability by directly influencing VEGFA levels. Therefore, we treated tube cells with exogenous TNF-α (47.5 pg/mL) and IL-6 (42.4 pg/mL) for 24 h to mimic the effects of 24-h exposure to 100 μg/mL DEPs. As seen in Fig 4A, the combination treatment with both cytokines for 24 h further increased the level of VEGF-A. Addition of Tin protoporphyrin IX (SnPP) (HO-1 inhibitor, 25 μM) might not affect the VEGF-A secretion. Quantification of the results is shown in Fig 4B, indicating that VEGF-A secretion was associated with a DEP-induced pro-inflammatory response but not directly correlated to HO-1.

On the other hand, our previous investigation suggested that DEPs upregulated HO-1 and induced VEGFA secretion in a dose-dependent manner. Moreover, Terry *et al* (1998 and 1999) suggested that TNF-α induces HO-1 expression in HUVEC monolayer cultures [42, 43]. Therefore, we further examined whether TNF-α + IL-6 would stimulate VEGF-A secretion through the same mechanism by measuring changes in HO-1 expression in this more biological relevant model following treatment with the cytokines. Here TNF-α plus IL-6 was added to endothelial tubes ± SnPP, and NAC + DEP for 24 h, and the level of HO-1 was assessed. Tube cells treated with 100 μg/mL DEP was used as a comparison. Interestingly, treatment of both cytokines did not change the level in HO-1 expression, but NAC significantly reduced DEP-upregulated HO-1 (Fig 4C). Quantification of these results is shown in Fig 4D. Moreover, as seen in Fig 4E, *HO-1* mRNA levels were not changed following exposure to TNF-α and IL-6.

### TNF-α and IL-6 induced vascular permeability independently of HO-1 activation

**Fig 5:**
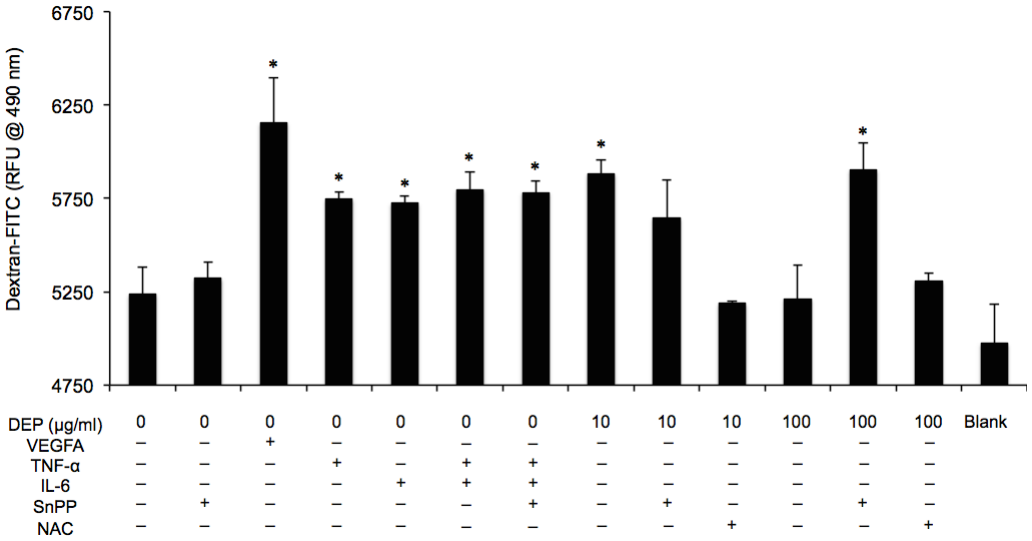
DEPs and the induced cytokines caused *in vitro* capillary permeability independently of HO-1 activation. FITC-dextran was added to endothelial tubes for 4 h after a 24-h exposure to different treatments. The RFU was determined for each sample after washing of the endothelial tubes.

Since TNF-α and IL-6 stimulated VPF/VEGF-A, further investigations were required to demonstrate a link with vasculature permeability. Although HO-1 has been shown to associate with VEGF-A-induced permeability, whether TNF-α and IL-6 are involved in the permeability mechanism of HO-1 activation remains unclear. To assess this issue, we evaluated the amount of dextran-FITC taken up by endothelial tubes exposed to TNF-α, IL-6, TNF-α plus IL-6 (with or without SnPP), and DEPs (10 or 100 μg/mL, with or without SnPP or NAC). Samples left untreated or treated with VEGF-A (100 ng/mL) were used as negative and positive controls, respectively. As shown in Fig 5, cytokine treatment induced changes in dextran uptake from 5237 ± 145 (RFU) (negative control) to 5747 ± 40 (TNF-α), 5723 ± 39 (IL-6), 5797 ± 95 (TNF-α plus IL-6), and 5781 ± 60 (TNF-α plus IL-6 and SnPP). Tube cell permeability induced by DEPs (10 μg/mL) was restored after the addition of SnPP or NAC. Moreover, 100 μg/mL DEPs and 100 μg/mL DEPs plus NAC reduced the amount of dextran-FITC taken up by the cells, while cotreatment with SnPP elevated dextran-FITC uptake. Although these values were not significantly different, there was a correlative trend between exposure of endothelial tubes to both cytokines and the amount of fluorescence taken up by the tubes, indicating changes in permeability.

### DEP-induced cytokines caused endothelial VE-cadherin redistribution

**Fig 6:**
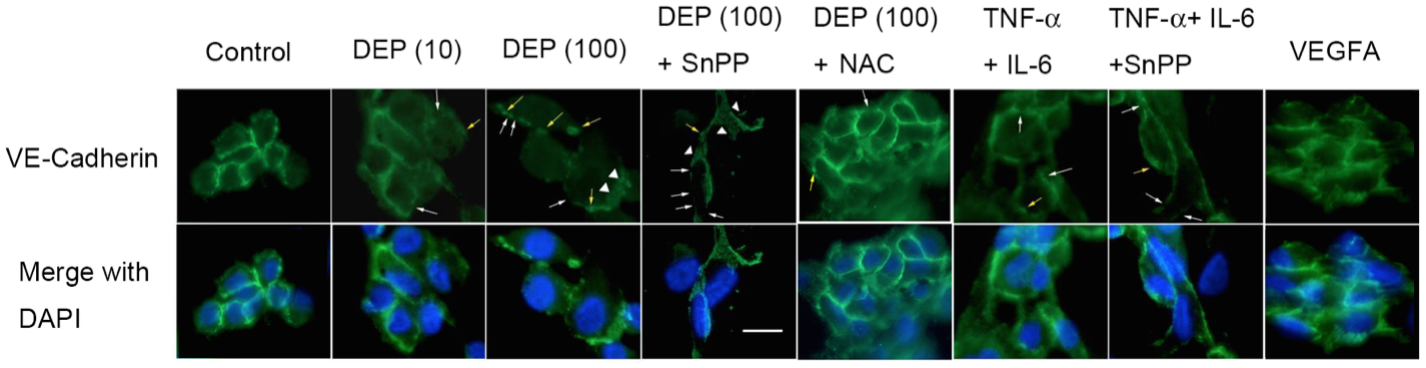
DEPs and the induced cytokines caused capillary VE-cadherin redistribution. Endothelial tube cells were treated for 24 h with DEPs±SnPP or TNF-α+IL-6± SnPP. An endothelial tube cell culture treated for 24 h with 100 ng/mL VEGFA was the positive control. VE-cadherin localization was then determined by epifluorescence imaging. White arrows indicate areas where VE-cadherin showed discontinuity around the cell membrane. Arrowheads indicate instances where VE-cadherin moved from the cell membrane to the interior and intracellular submembrane regions. Yellow arrows indicate cell-cell separations and globules of VE-cadherin that accumulated intracellularly near the cell membrane.

In contrast to VEGFA-dependent permeability, VE-cadherin-dependent cell-cell barriers are crucial to preventing the permeability of endothelial cells [8, 44–46]. The effects of TNF-α and IL-6 on VE-cadherin distribution in endothelial tube cells are shown in Fig 6. Discontinuity of VE-cadherin localization on the cell-cell border was observed (white arrows), and some VE-cadherin internalization was present, as indicated by punctate staining in the cytoplasm (triangular arrows). Some VE-cadherin was pulled from the cell surface into globules under the cell membrane (yellow arrows). To confirm whether TNF-α and IL-6 had the ability to rearrange the VE-cadherin pattern without HO-1 activation, cells were exposed to cytokines plus SnPP. Furthermore, to assess whether NAC restore the VE-cadherin redistribution, NAC was used with DEPs (10 or 100 μg/mL). VEGF-A was used as controls. Interestingly, endothelial tube structures were altered with increasing concentration of DEPs. At 100 μg/mL, cells were rounded up, and pulling and thinning of the cytoplasm between the cells were observed. Additionally, redistribution of the globules of submembrane VE-cadherin was found, while little discontinuity of VE-cadherin was observed at 10 μg/mL. Additionally, most of the cells exhibited regular separations from each other, and cells exposed to TNF-α and IL-6 along with the HO-1 inhibitor SnPP exhibited strong distribution of VE-cadherin on the cell border, indicating reduced permeability. These data suggested that TNF-α+IL-6 may contribute to the permeability of cell junctions, similar to VEGF-A exposure.

### DEP-caused VEGFR2 rearrangement

**Fig 7:**
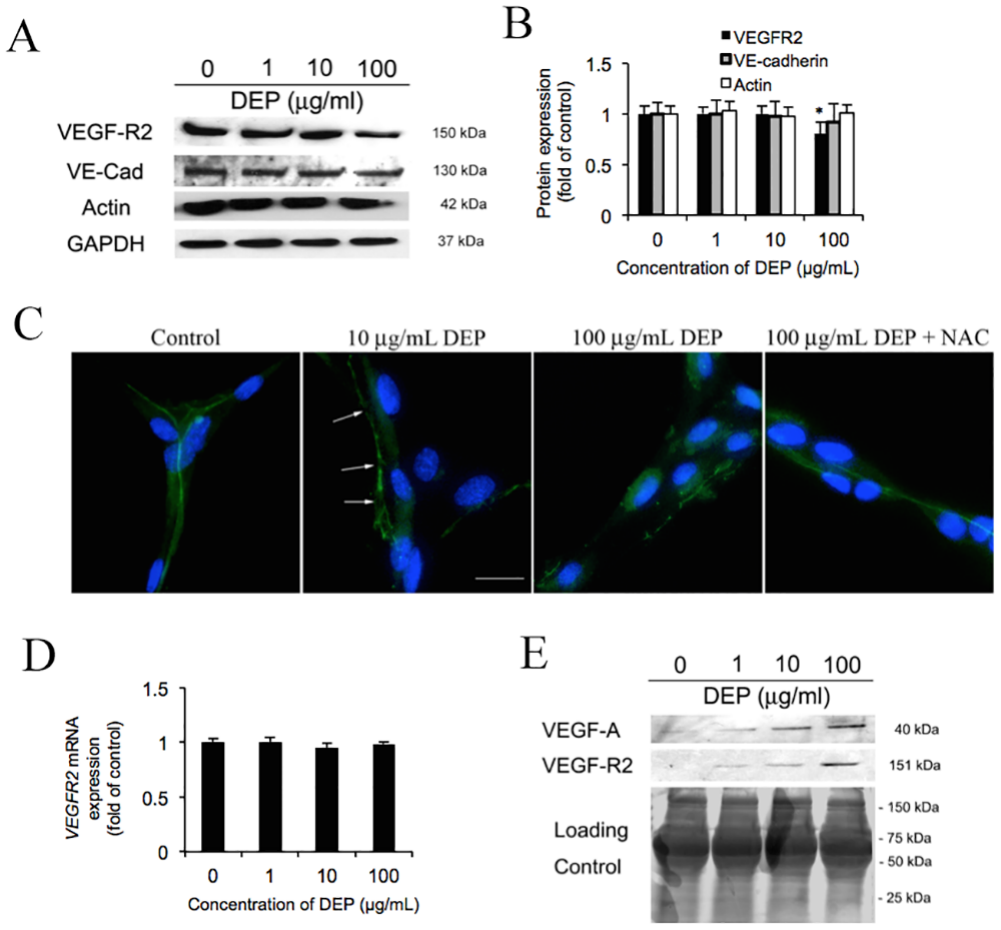
DEP dissociate VEGF-R2 from adherens junction connection. (A) After 24 h DEP (1, 10 and 100 g/mL) exposure, the tube cells were lysed and harvested. The untreated cells (0 g/mL) were defined as negative control. The level of adheren junctional proteins VEGF-R2, VE-cadherin (VE-Cad), -catenin and cytoskeleton actin were determined by Western blot. Equal amount of protein loading was confirmed based on total GAPDH expression. (B) Quantification showed VEGF-R2 decrease in a dose dependent manner as the relative fold change to the control. Again, the level of VE-cadherin, -catenin and cytoskeleton actin activities are unaffected by DEP. (C) Images show the distribution of VEGF-R2 in response to DEP (10 and 100 g/mL) and DEP (100 g/mL) plus NAC exposure for 24 h. Nuclei were stained with 10 mM DAPI. Shown is a representative image from three independent experiments. Scale bar = 10 m. Magnification is 400X. (D) To confirm whether DEP change *VEGF-R2* expression, the cDNA were probed with VEGFR2 primers and the activity were analyzed using QPCR after exposure with DEP for 24 h. The level of mRNA normalized to 18s gene expression was presented as the fold change relative to the untreated control (0 μg/mL). Means ± SD, n = 3. (E) Effects of DEP on the secretion of VEGF-R2. Secretion of VEGF-R2 of capillary tube cells related to the DEP exposure. After 24 h DEP (1, 10 and 100 g/mL) exposure, the supernatant/medium were collected and loaded (40 L/well) onto the SDS-PAGE. The expression of secreted VEGF-R2 was tested and measured by using western blot. Coomassie blue stain was defined as the loading control. The supernatant collected from the cells treated without DEP were defined as negative control.

The cytoplasmic tail of VE-cadherin is known to interact with VEGF-R2 and β-catenin and form a complex linking the actin cytoskeleton to the cell membrane to stabilize cell shape and adherens junctions [47]. Depolarization of actin cytoskeleton network and disruption of VEGF-R2-VE-cadherin formed complex might not only affect endothelial cell-cell contact, but also regulate cell survivability [12, 47–49]. Hence, here we assess whether VEGF-R2 and other junctional proteins change in response to DEP. As shown in Fig 7A, VE-cadherin and actin are unaffected by DEP exposure, but only VEGF-R2 is decreased slightly. According to a quantitation analysis (Fig 7B), VEGF-R2 expresses at 87.5% and 75% intensity of control at 10 and 100 μg/mL respectively, but remains unchanged at 1 μg/mL. Furthermore, fluorescence images show the morphology of VEGF-R2 that is outlining the cell junctions fairly regularly in the negative control. Image of DEP (10 μg/mL) treated tube cells indicate a loss in sharpness of border (arrows) to the VEGF-R2 staining. At 100 μg/mL DEP treated tube cells, which have a similar VEGF-R2 performance to 10 μg/mL, but has more discontinuity patterns on the cell-cell boarder. Addition of NAC mitigates this VEGF-R2 alternation. On the other hand, our qPCR result reveals that *VEGF-R2* mRNA level is unaffected (Fig 7D). This interesting result implies that VEGF-R2 may be secreted to the supernatant/medium. As shown in Fig 7E, western blot indicated that sVEGF-R2 (soluble VEGF-R2) and VEGF-A in the medium are dramatically increased in a DEP dose dependent manner.

## Discussion

The cellular production of ROS is a well-known physiological process. Several studies have provided evidence that free radical-induced oxidative damage of cell membranes, DNA, and proteins might be the cause of several diseases including cardiovascular events [50, 51]. In previous studies, we have proposed and supported the hypothesis that DEPs affect vascular permeability through VPF/VEGF-A release by production of ROS. This has been demonstrated by experiments showing that networks of the adherens junctional protein VE-cadherin become disrupted when cultured cells are treated with DEPs [8]. In the current work, we performed a more extensive characterization of the pro-inflammatory response to DEP exposure. The goals of this study were to investigate the induction of extracellular ROS generation, secretion of the cytokine-generating moiety, the mechanism leading to increased vascular permeability, and the influence of VE-cadherin relocalization, and the mitigation of NAC for these disorders described above. We also sought to clarify whether a system without the confounding effects of inflammatory cells could be used to examine how DEPs might modulate endothelial permeability.

Three different methods have been used to demonstrate that DEP exposure is able to generate oxidative stress [12]. However, whether these free radical species are generated by DEPs themselves, before they contact the cell/the inside of the cell, or after they are taken up remains unclear. Therefore, we evaluated both extracellular and intracellular ROS with the Cm-H_2_DCFDA assay (Fig 1A and 1B). Interestingly, only H_2_O_2_ sample increased ROS levels following a 2-h incubation, and DEPs did not induce extracellular ROS. However, in contrast to this cell-free result, Cm-H_2_DCFDA assays showed that DEP induced intracellular ROS generation in a dose-dependent manner in tube cells. Furthermore, the cytotoxicity curves showed a pattern that was quite similar to the intracellular ROS data, implying that DEP-induced intracellular ROS should be a factor in determining cell survival. These experiments not only confirmed the results reported by Chao *et al* (2012), but also provided direct evidence that DEPs induced cytotoxicity through generation of free radicals in tube cells [12].

Exposure to DEP causes substantial changes in cellular antioxidant/oxidant parameters, and this phenomenon is the predominant evidence of a shift in the redox equilibrium towards oxidation [52–54]. After 24 h of supplementation, NAC provided remarkable changes in antioxidant/oxidant status of the DEP treated tube cells including extracellular and intracellular ROS (Fig 1A and Table 1). Cellular thiols play central roles in maintaining the cellular redox status [50, 51, 55]. The increases observed in total GSH levels might be an adaptive response toward an oxidative insult. The elevation in GSSG levels indicates the ability of DEP to interfere with GSH and cause its oxidation. In addition, GSH/GSSG redox ratio decreased significantly with DEP treatment. Administration of NAC with DEP exposure provided significant increases in the redox ratio (Table 1). Recently, we showed that NAC protects *in vitro* AS52 CHO cells from the oxidative damage caused by 3,5-dimethylaminophenol [37]. It is well known that •OH is formed from H_2_O_2_ and it is the most detrimental ROS, due to its high interaction with nucleic acids, proteins, and lipids. NAC terminates these chain radical reactions by transferring a single electron, owing to the stability of its own radical ion. Thus, the results suggest that NAC might suppress DEP-induced ROS efficiently and reduce sequential damages due to its effect on cellular redox equilibrium [56].

Promotion of inflammation is considered a key step in the adverse health effects associated with PM exposure [57, 58]. Additionally, DEPs cause the release of pro-inflammatory cytokines (e.g., TNF-α, IL-6, and IL-1β) both *in vitro* and *in vivo* [27, 59–63]. Our results showed that intracellular ROS were also able to contribute to the release of pro-inflammatory cytokines (Fig 2). However, only TNF-α and IL-6 were secreted from the capillary-tube cells following 24-h exposure to DEPs. No IL-1β was detected, even at the highest dose of DEP, although many studies in monolayer endothelia have demonstrated the opposite results [43, 64–66]. Our immunoblotting results also confirmed this result. Krishnan *et al* (2013) suggested that IL-1β might be increased within 7 h after inhalation of diesel exhaust (DE) [67], and the inactive proform of IL-1β may be cleaved rapidly, enabling an immediate response to occur [27]. These reasons may explain the lack of IL-1β expression in our experiment.

Obviously, VEGF-A promotes vascular permeability [68–71]. In addition, TNF-α and IL-6 have been shown to stimulate permeability; however, this effect has only been observed in endothelial monolayer culture [29, 31]. Therefore, we sought to identify the mechanisms mediating vascular permeability in our model (Fig 5). Our data demonstrated that both cytokines were independently capable of enhancing VEGF-A secretion in the absence of DEPs. While the effects were highest at 24 h, no significant difference was observed at different time points. In addition, VEGFA is modulated by HO-1 during the stimulation of vascular permeability [18, 72, 73]. Our previous research also supported the link between HO-1 and VEGF-A secretion in response to DEP exposure and demonstrated that VEFG-A levels were reduced by the addition of NAC or SnPP [12]. Although the functions of TNF-α and IL-6 have been investigated widely, the mechanism through which they contribute to vascular permeability following HO-1 upregulation is still unknown. Therefore, we treated the tube cells with TNF-α+IL-6±SnPP and compared the results to those of samples exposed to DEP. While DEP induced HO-1 expression, no significant differences were detected among samples treated with TNF-α+IL-6+SnPP, TNF-α+IL-6, and the control. Additionally, we found that *HO-1* mRNA was not altered in response to treatment with any of these cytokines, suggesting while TNF-α and IL-6 elevated VEGF-A, they did not enhance HO-1 expression. This may be because endothelial permeability was facilitated by ROS-dependent induction of TNF-α and IL-6, which possibly upregulated VEGF-A independently of HO-1 stimulation. However, times shorter than 24 h have not been tested.

VEGF-A can mediate several signaling pathways [20], including endothelial permeability, as shown in our study. Although lacking a standard method to assess the permeability of capillary-like tubes, we followed our previous method and evaluated whether dextran was taken up by endothelial tubes exposed to different treatments for 24 h [12]. Additional exogenous VEGF-A (100 ng/mL) applied to the culture medium as the positive control increased permeability in tube cells. Obviously, VEGF-A, DEPs, and DEP-induced TNF-α and IL-6 also markedly increased vascular permeability in Fig 5. However, no significant differences were observed between these treatments. Importantly, the use of NAC attenuated DEP-induced ROS generation, with levels of ROS reaching those of the negative control, suggesting that no free radicals, TNF-α, IL-6, or VEGF-A were generated. However, application of the HO-1 inhibitor SnPP concurrent with DEPs slightly restored permeability, possibly because some TNF-α (22.7 pg/mL) and IL-6 (21.8 pg/mL) remained in the medium, affecting permeability. Increasing concentrations of DEPs correlated with small increases in dextran-FITC uptake, with the exception of the 100 μg/mL DEP sample, although we have shown that cells undergo rounding, with pulling and thinning of the cytoplasm at this highest dose [8]. This is likely because high concentrations of DEPs (e.g., 100 μg/mL) caused massive cell death (over 50%), destroying the cell-cell network of adhesive contacts; otherwise, we would expect the value to be quite high. Interestingly, cotreatment with SnPP restored the values to that of the positive control, indicating that TNF-α and IL-6 may function independently of HO-1 to induce VEGF-A release and endothelial permeability.

Furthermore, we found that the localization of VE-cadherin to the endothelial cell-cell border became disrupted, with highly irregularly shaped cells displaying large gap formations, suggesting that the permeability of the endothelial layer was increased in response to cytokines. These results were consistent with our observation that TNF-α and IL-6 induced VEGF-A expression to stimulate vasculature leakiness. Although the localization patterns of VE-cadherin included linear discontinuity and globular formation, no dramatic differences in the shape were detected between exposure to DEPs (10 μg/mL), TNF-α+IL-6, and TNF-α+IL-6+SnPP. At 100 μg/mL DEPs, VE-cadherin was internalized in endothelial tube cell cultures, causing cell-cell gaps that led to vascular permeability, NAC is able to entirely restore these alternations. Co-treatment of SnPP with DEP (100 μg/mL), VE-cadherin still exhibited irregular patterns of localization in the cytoplasm; however, the cell-cell border was protected and detectable on the cell membrane in a similar manner to the TNF-α plus IL-6-treated sample. This result suggested that vascular permeability might be stimulated by TNF-α and IL-6 independently of HO-1 activation.

VE-cadherin limits cell proliferation and form a cell-cell barrier by retaining VEGF-R2 at the membrane and preventing its relocation into signaling compartments [12, 47–49]. VEGF-R2 is portable and its mechanism remains unclear, although Dajana (2004) mentioned that VEGF-A binds to VEGF-R2 for either stabilization in confluent endothelial cells or proliferation in sparse endothelial cells [44]. Meanwhile, *in vitro* and *in vivo* studies have demonstrated that soluble VEGF-R1 can be secreted for the binding of VEGF-A and results in prohibition of angiogenesis [74]. However, it is unclear how dissociated VEGF-R2 plays a role in the physiological / pathological function during exposure to DEP. The signalling cascade stimulated by VEGF-A activates VEGFR-2 followed by c-Src tyrosine kinase and MAP kinases the Erk subfamily activation. The consequent effect has been suggested to disrupt endothelial cell-cell junctions leading to restrict an increase in vascular permeability to the environment affected by local injury to blood vessels [75]. This might lead to cause acute or chronic cardiovascular disorders including peripheral edema, capillary degradation, and inflammation [76–78]. Here we propose that the VE-cadherin/VEGF-R2 complex is disrupted, causing the tube cells to lose their cellular contact, and the dissociated VEGFR2 may be released and bind to the VEGFA in the medium for reduction of the increase vascular permeability.

## Conclusion

**Fig 8:**
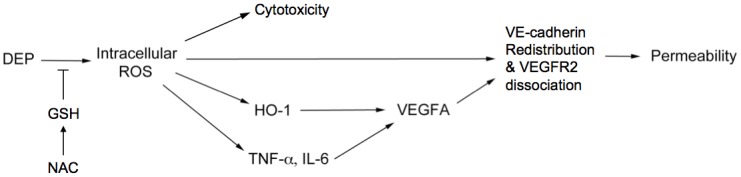
Schematic diagram depicting DEP-induced mechanisms potentially causing vascular permeability. DEPs cause capillary-like endothelial tubes to produce intracellular oxidative stress, which increases vascular permeability by 3 mechanisms: i) direct rearrangement of the cell-cell junctional VE-cadherin network; ii) activation of HO-1, which subsequently causes release of the vascular permeability factor, VEGFA; iii) inducible TNF-α and IL-6 release, which might increase the release of VEGFA independently of the contribution by HO-1. Administration of NAC blocks these sequential cytotoxic effects from the beginning.

To our knowledge, this is the first study showing that antioxidant, NAC is capable of reducing the oxidative increase of vascular permeability exerted by diesel exhaust particles. There was a common relationship between the effects of DEP on GSH homeostasis in that was positively affected [79, 80]. Our previous experiments have shown that DEPs upregulate HO-1 expression and sequentially induce the release of VEGF-A. Both of these molecules are known to contribute to endothelial permeability. The current study shows that DEP-induced intracellular ROS may cause the release of pro-inflammatory TNF-α and IL-6, which may induce endothelial permeability by promoting VEGF-A secretion independently of HO-1 activation. A schematic depicting how DEPs affect permeability is provided in Fig 8. In this figure, the key mediator is intracellular ROS induced by DEP exposure. The ROS is cytotoxic to the tube cells, while NAC is able to mitigate this free radicals generation. Chao et al (2012) has suggested this intracellular ROS directly increases vascular permeability and stimulates Nrf2 translocation to the nucleus, thereby upregulating HO-1 expression to modulate VEGF-A secretion [12]. Alternatively, intracellular ROS-induced TNF-α and IL-6 contribute to the release of VEGF-A as well. These 3 pathways lead to disruption of the endothelial VE-cadherin network. The pattern of changes in VE-cadherin distribution contains linear discontinuity, submembrane internalization, and globular formation, resulting in discontinuation and permeability of the cell-cell border. Therefore, were these phenomena to occur, even to a small degree, in lung capillaries after exposure to DEP, the particles may gain access to the bloodstream and contribute to the development of adverse cardiovascular events. And a potential protection of NAC against the DEP-induced subsequent damages was determined.
